# A Toolbox for Herpesvirus miRNA Research: Construction of a Complete Set of KSHV miRNA Deletion Mutants

**DOI:** 10.3390/v8020054

**Published:** 2016-02-19

**Authors:** Vaibhav Jain, Karlie Plaisance-Bonstaff, Rajnikumar Sangani, Curtis Lanier, Alexander Dolce, Jianhong Hu, Kevin Brulois, Irina Haecker, Peter Turner, Rolf Renne, Brian Krueger

**Affiliations:** 1 Department of Molecular Genetics and Microbiology, University of Florida, Gainesville, FL 32610, USA; vaibhavjain@ufl.edu (V.J.); karliebonstaff@gmail.com (K.P.-B.); rsangani@gru.edu (R.S.); laeucu@ufl.edu (C.L.); adolce@ufl.edu (A.D.); jianhong.hu@bcm.edu (J.H.); irina25@me.com (I.H.); pturner@mgm.ufl.edu (P.T.); brian.j.krueger@gmail.com (B.K.); 2 Department of Molecular Microbiology and Immunology, Keck School of Medicine, University of Southern California, Los Angeles, CA 90033, USA; kbrulois@stanford.edu; 3 UF Health Cancer Center, University of Florida, Gainesville, FL 32610, USA; 4 UF Genetics Institute, University of Florida, Gainesville, FL 32610, USA

**Keywords:** KSHV, human herpesvirus 8, miRNA, bacmid

## Abstract

Kaposi’s sarcoma-associated herpesvirus (KSHV) encodes 12 viral microRNAs (miRNAs) that are expressed during latency. Research into KSHV miRNA function has suffered from a lack of genetic systems to study viral miRNA mutations in the context of the viral genome. We used the *Escherichia coli* Red recombination system together with a new bacmid background, BAC16, to create mutants for all known KSHV miRNAs. The specific miRNA deletions or mutations and the integrity of the bacmids have been strictly quality controlled using PCR, restriction digestion, and sequencing. In addition, stable viral producer cell lines based on iSLK cells have been created for wildtype KSHV, for 12 individual miRNA knock-out mutants (ΔmiR-K12-1 through -12), and for mutants deleted for 10 of 12 (ΔmiR-cluster) or all 12 miRNAs (ΔmiR-all). NGS, in combination with SureSelect technology, was employed to sequence the entire latent genome within all producer cell lines. qPCR assays were used to verify the expression of the remaining viral miRNAs in a subset of mutants. Induction of the lytic cycle leads to efficient production of progeny viruses that have been used to infect endothelial cells. Wt BAC16 and miR mutant iSLK producer cell lines are now available to the research community.

## 1. Introduction

**Figure 1:**
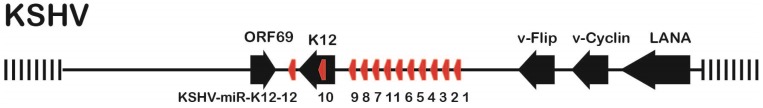
KSHV Latency-Associated Region (KLAR) showing open reading frames (black arrows) and miRNAs (red arrows). The region between LANA and K12 on the minus strand is located between nts position 129,256 (LANA ATG) and 117,738 (K12 UGA) on the KSHV Bac16 genome (Accession number GQ994935).

Kaposi’s sarcoma-associated herpes virus (KSHV) is the etiological agent causing Kaposi’s sarcoma (KS), primary effusion lymphoma (PEL) and a subset of multicentric Castleman’s disease (MCD) [1,2,3]. KSHV, also named Human Herpesvirus Type 8 (HHV-8), is a γ-herpesvirus which significantly contributes to mortality of HIV-infected individuals or patients undergoing organ transplants [3]. Most cells in KSHV-associated malignancies are latently infected and express only a very limited number of viral genes, which contribute to viral pathogenesis and/or tumorigenesis by targeting a number of host regulatory pathways (reviewed by [4]). In addition to four major latency associated protein-encoding genes (LANA, v-FLIP, v-Cyclin, and the Kaposin family of proteins), the major KSHV latency-associated region (KLAR) also encodes 12 microRNA (miRNA) genes (Figure 1).

MiRNAs are short non-coding RNAs that post-transcriptionally regulate gene expression predominantly through binding to the 3'UTRs of mRNAs which leads to translational inhibition or mRNA degradation [5,6]. After the initial discovery of virally-encoded miRNAs in EBV in 2004 [7], several laboratories [8,9,10,11] identified 12 miRNA genes that theoretically can give rise to 25 mature miRNAs in lymphoma cells latently infected with KSHV. However, how many KSHV miRNAs are expressed at functionally relevant levels is not clear, and might also be cell-type specific [12]. The discovery of γ-herpesvirus-encoded miRNAs has created a novel field of intense study partly because of the attractive hypothesis that miRNAs expressed during latency, while potentially having profound effects on host cellular gene expression, would not elicit adaptive immune responses. To date, a large number of studies have reported viral miRNA/mRNA target interactions for either viral or host cellular genes, and much of these data suggest that KSHV miRNAs target a number of central signal transduction pathways as well as host and/or viral genes controlling latency/lytic viral replication. However, very few studies have attributed phenotypes to the presence or absence of specific miRNAs within the context of viral infection (for review see [13,14]).

Recent comprehensive target analysis for KSHV-encoded miRNAs using ribonomics approaches revealed nearly 1000 potential target genes, pointing to central pathways, such as apoptosis, cell cycle control, innate immune responses, and carbohydrate metabolism, to name a few, which all likely contribute to KSHV pathogenesis and/or tumorigenesis [15,16,17,18]. It is important to note that these data have been generated from latently-infected primary-effusion lymphoma cells, originally isolated from HIV-positive patients. Since mammalian miRNAs function in a cell-type specific manner, it will be crucial to analyze the contribution of viral miRNAs during early events of infection in different cell types that are relevant to KSHV biology (*i.e.*, B cells, dendritic cells, endothelial, and epithelial cells). To address this, we created a complete set of viral mutants that each lack one specific miRNA or express no miRNAs at all. We utilized KSHV BAC16, which was developed and fully sequenced in the Jung laboratory [19,20] and created stable latently-infected KSHV producer cell lines from each mutant bacmid on the background of iSLK cells developed in the Ganem laboratory. iSLK producer cells contain a tet-inducible RTA and after induction give rise to high titer progeny virus, which can be used in *de novo* infection studies [21]. This mutant collection, which is quality controlled by whole-genome sequencing, provides standardized tools which should accelerate our understanding of how KSHV-encoded miRNAs function in viral biology and pathogenesis.

## 2. Materials and Methods

More detailed protocols including step-by-step procedures for bacmid mutagenesis in *Escherichia coli*, and recovery of infectious KSHV virus in mammalian cells, are available as supplemental files.

### 2.1. Cell Lines

293T cells are human embryonic kidney (HEK) cells transformed with large T antigen of SV40. iSLK cells used to create virus producer cell lines were a generous gift from Don Ganem [21]. TIVE cells are Telomerase Immortalized Vein Endothelial cells described in [22].

### 2.2. Mutagenesis Strategy

**Figure 2:**
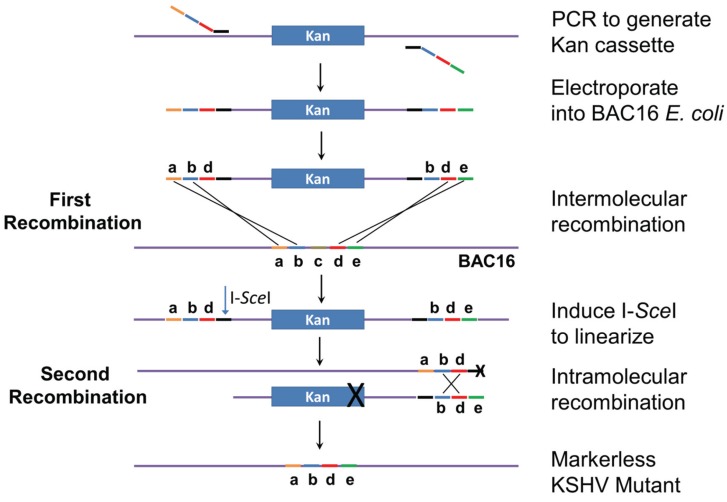
Two-step recombineering. Primers are designed to PCR amplify the kanamycin-resistance cassette. The primer tails match KSHV sequences (*abd* for the forward primer and *bde* for the reverse primer). Note that sequence *c* will be deleted in the final construct. The PCR product is electroporated into *E. coli* cells carrying BAC16, and intermolecular recombination generates a kan-resistant bacmid. Induction of I-*Sce*I results in linearization of the bacmid. Intramolecular recombination between sequences *bd* near the ends of the molecule produces the final markerless KSHV mutant bacmid, with sequences *abde*. Modified from [23,24].

The genomic locations and sequences of the 12 KSHV miRNA genes are shown in Figure 1 and Table S1. MiRNA deletions and mutations were constructed in *E. coli* GS1783 which is a recA^−^ recombination-deficient strain containing the Red recombination system. Mutagenesis is performed as a two-step process as initially described [23,24]. In the first step (Figure 2), a mutation cassette containing a selectable marker (kanamycin) is created by PCR and recombined into the KSHV genome at the exact position where the deletion is to occur. In the second step, intra-molecular recombination is facilitated by a duplicated sequence flanking the mutagenesis cassette which results in the removal of the positive selection marker and the deletion of the target sequence. For the deletions, primers were created with sequences flanking the region targeted for deletion. Primer sequences are listed in Table S2. The forward primer contained 40 bases of sequence upstream of the deletion, 20 bases downstream of the deletion, and 20 bases of sequence corresponding to the selectable marker. Reverse primers were similar except they contained 20 bases from the selectable marker, 20 bases of genomic sequence upstream of the deletion followed by 40 bases of sequence corresponding to the region downstream of the deletion. For the insertion mutation of miR-K12-10, which is embedded in the K12 (kaposin) ORF, an additional 23 base region was added in between the upstream and downstream regions of the forward and reverse primers. This insertion disrupted the stem loop without affecting the K12 ORF, however the editing of both the K12 mRNA (and miRK12-10) miRNA is interrupted by this strategy [10,25].

After PCR amplification, the mutation cassette was introduced into *E. coli* GS1783 containing the inducible Red recombination system and wt KSHV BAC16 [20], which contains the bacmid backbone pBelo45 inserted between ORF57 and K9 (vIRF1) (generously provided by Jae Jung, University of Southern California, Los Angeles, CA, USA). Bacteria were grown to an O.D. of 0.5 and Red recombinase expression was induced by temperature shift to 42 °C for 10 min. To generate electrocompetent *E. coli* GS1783, cells were chilled on ice for 10 min and washed four times with ice cold ddH_2_O. The amplified mutation cassette (100 ng) was electroporated using a Bio-Rad gene pulser XCell set to 1.5 kV, 25 μF, and 200 Ω. After incubation in SOC media at 37 °C bacteria were plated on kanamycin plates.

The clones created in the first step of the recombination procedure were then screened to determine if they contained the positive selection marker. Colony PCR was performed using a primer corresponding to a region upstream of the region to be deleted and a primer at the end of the positive selection marker. This PCR strategy serves two purposes, it shows that the positive selection marker is present in *E. coli* and, by spanning both the viral genome and the positive selection marker, it shows that the recombination cassette has likely properly recombined with the bacmid. Since the Red recombination system relies on using repetitive sequences to perform the mutation, the terminal repeats (TRs) of the KSHV genome can easily be shortened or unintentionally mutated. To prevent this, all mutant bacmids were digested by NheI, which does not cut in TR sequences, and analyzed by pulsed field gel electrophoresis (PFGE). Only clones with wt BAC16 TR length were used for subsequent recombination steps.

For the second step of Red recombination, the bacmid-containing bacteria were grown to an O.D. of 0.5 and then I-SceI expression was induced by the addition of arabinose to a final concentration of 2% and incubation for 45 min. I-SceI linearizes the bacmid to prepare it for intramolecular recombination which results in the removal of the positive selection marker and the generation of a marker-less viral mutant. This is accomplished by the inclusion of an I-SceI restriction site in the forward primer used to generate the mutation cassette (Figure 2). After I-SceI induction, the *E. coli* was incubated for 10 min at 42 °C to induce the expression of Red recombinase. The cells were incubated for 1 h at 32 °C and then 10-fold serial dilutions were plated.

After the second round of recombination, colonies were streaked on replica plates containing either chloramphenicol or the positive selection marker kanamycin to screen for clones that successfully removed the positive selection marker and likely contained the proper mutation. The final mutants were verified as follows. First, colony PCR was performed to show that the positive selection marker was lost from the viral bacmid genome. The integrity of the terminal repeats was determined by PFGE as described above. To show that the target region was deleted, PCR products spanning the region to be deleted were run on TBE 6% acrylamide gels. If the target region was correctly deleted, the product would be 60 bp in length compared to the 80 bp wt band.

### 2.3. Co-Culture Infection of iSLK Cells

In order to produce recombinant KSHV virus, bacmid DNA was transfected into 293T cells using TransIT-293 Transfection Reagent (Mirus, Madison, WI, USA). Cells were seeded in six-well plates and transfected with 2 μg of bacmid DNA the next day. Transfection efficiency was determined by observing GFP expression 48 h post transfection. The highest transfection efficiency, between 40%–60%, was seen when using freshly prepared bacmid DNA along with newly-thawed 293T cells. Next transfected cells were transferred to a 10 cm dish containing media supplemented with 100 μg/mL hygromycin B to select for bacmid containing cells. 293T cells were allowed several days to recover from antibiotic selection and expanded to 15 cm plates. This process usually takes 10–15 days. Once 293T/Bac cells were 40%–60% confluent, lytic replication was induced by the addition of 20 ng/mL TPA, and 1 mM Valproic acid. The induced cells were trypsinized and replated with an equal number of iSLK cells. Infected iSLK cells were selected by using 1200 µg/mL Hygromycin and 10 µg/mL puromycin, which counterselects against 293T cells. 100% Confluent iSLK cells were expanded, cryopreserved and used for further quality control experiments, such as whole genome Illumina sequencing and miRNA expression analysis by TaqMan assay.

### 2.4. Isolation and Quantification of Recombinant Virus from iSLK Cells

Filtered media from induced iSLK cells was subjected to ultracentrifugation through a 25% sucrose cushion at 100,000× *g* for 1 h. Virus pellets were resuspended in 1% of original volume using serum free media and stored at −80 °C. DNA from 25 μL of recombinant virus stocks was isolated using DNAzol (Life Technologies, Carlsbad, CA, USA), according to the manufacturer’s recommendations. Virus DNA was resuspended in 30 μL of ddH_2_O and 1 μL was used per qPCR reaction. Real-time qPCR was performed using pcDNA3.1-ORF73 plasmid as a standard along with primers specific for the N-terminus of LANA (ORF73). The viral genome copy number was determined by comparing viral DNA to the plasmid standard curve.

### 2.5. TaqMan Assays

To confirm lack of miRNA expression in recombinant virus, we used TaqMan miRNA RT-qPCR assays (Life Technologies). RNA from 293T cells transfected with KSHV Bacmid DNA was isolated using RNA-Bee (Tel-Test, Friendswood, TX, USA) according to the manufacturer’s suggestions. 10 ng of total RNA was used in the miRNA reverse transcription reaction. Stem loop primers specific for all 12 KSHV-miRNAs along with control RNU66 were used to make cDNA pools. TaqMan miRNA qPCR was performed using cDNA with specific primers and probes for each KSHV-encoded miRNA (ABI), together with an RNU66 endogenous control.

### 2.6. Illumina Sequencing and Enrichment of Viral Genomes Using SureSelect System

To ensure that no unintended mutations were created, we performed whole genome sequencing of viral episomes in latently-infected iSLK cells. The biotin-labeled RNA baits specific for KSHV genome were designed with eArray XD (Agilent, Santa Clara, CA, USA) with help from Agilent. The RNA baits are 120 nt long with 4× tiling frequency. Sequencing libraries were constructed from KSHV-infected cells as previously described [26]. After adapter ligation, DNA fragments within range of 150 bp to 300 bp were gel-selected and amplified with 10 cycles in first PCR. Samples were purified with Agencourt AMPure XP beads (Beckman Coulter, Brea, CA, USA). The KSHV-specific DNA sequences were enriched with SureSelect Enrichment System (Agilent) according to the manufacturer’s instruction. The first purified PCR products were denatured and hybridized with KSHV RNA baits at 65 °C for 48 h in PCR machine with heated lid. The RNA-DNA hybrids were recovered with Dynal MyOne Streptavidin T1 magnetic beads (Invitrogen, Carlsbad, CA, USA). The captured DNA was eluted and purified. The DNA was re-amplified with 22 cycles in PCR with primers (5′-AATGATACGGCGACCACCGAGATCTACACTCTTTCCCTACACGACGCTCTTCCGATCT and 5′-CAAGCAGAAGACGGCATACGAGCTCTTCCGATCT), and purified using Agencourt AMPure XP beads. The libraries were quantified with QuantIT dsDNA Assay Kit (Invitrogen) and sequenced with Genome Analyzer IIx (Illumina, San Diego, CA, USA). Using barcoding, four mutants were sequenced per lane at a read length of 55 nts in paired end mode. Sequencing data was analyzed using CLC Genomics Workbench (Qiagen, Redwood City, CA, USA).

## 3. Results and Discussion

### 3.1. Mutation Strategy

The locations of the KSHV miRNAs miR-K12-1 through miR-K12-12 with respect to ORFs in the KLAR (KSHV latency associated region) are shown in Figure 1. The sequences of KSHV miRNAs miR-K12-1 through miR-K12-12 are listed in Table S1, together with the nucleotide coordinates within KSHV BAC16 (Accession number GQ994935), and alternative names for the miRNAs in earlier publications [8,9,10,11].

To generate a complete set of miRNA mutations, we used BAC16 [19,20] in combination with the two-step Red recombinase system in *E. coli* GS 1783 [23,24]. BAC16, which contains pBelo45 backbone inserted between the KSHV vIRF-1 and ORF57 genes of the KSHV JSC-1 strain [27], is genetically stable and, based on whole-genome sequencing, does not contain duplications or deletions as was recently reported for BAC36 [20,28]. To create 11 of the 12 specific microRNA mutants, 20–25 bp long regions were deleted from one arm of each pre-miRNA hairpin. To ensure that no alternative hairpin structures were created, RNA-fold was applied to all mutant sequences plus minus 100 bp of the deletions. The strategy to only delete 20 nts within one miRNA arm should effectively destroy the formation of the pre-microRNA hairpin without grossly affecting the expression of neighboring microRNAs within the cluster. This strategy also eliminates the need to delete both 5p and 3p microRNA regions since the target of the mutation is not the DNA sequence itself, but the dsRNA which gets processed by Drosha. The sequences of the primers used to create the individual miRNA deletions are listed in Table S2, together with the coordinates in BAC16 (Accession number GQ994935) of the deleted regions. In the case of miR-K12-10, which is embedded within the kaposin (K12) open reading frame, a total of eight point mutations were introduced within the microRNA to disrupt hairpin formation while preserving the protein coding sequence of Kaposin (Table S2). To delete the miRNA cluster containing 10 pre-miRNAs a 2.2 kbp deletion was created (ΔmiR-cluster). Starting with ΔmiR-cluster, a mutant lacking all miRNAs (ΔmiR-all) was created by consecutively mutating the two remaining miRNAs (miR-K12-10 and miR-K12-12) located outside of the cluster.

### 3.2. Generation of Recombinant KSHV Bacmids and Quality Control

The steps for generating recombinant bacmids harboring individual miR mutations are shown in Figure 2. The procedure is described in Materials and Methods and more fully in Supplementary Protocol 1.

**Figure 3:**
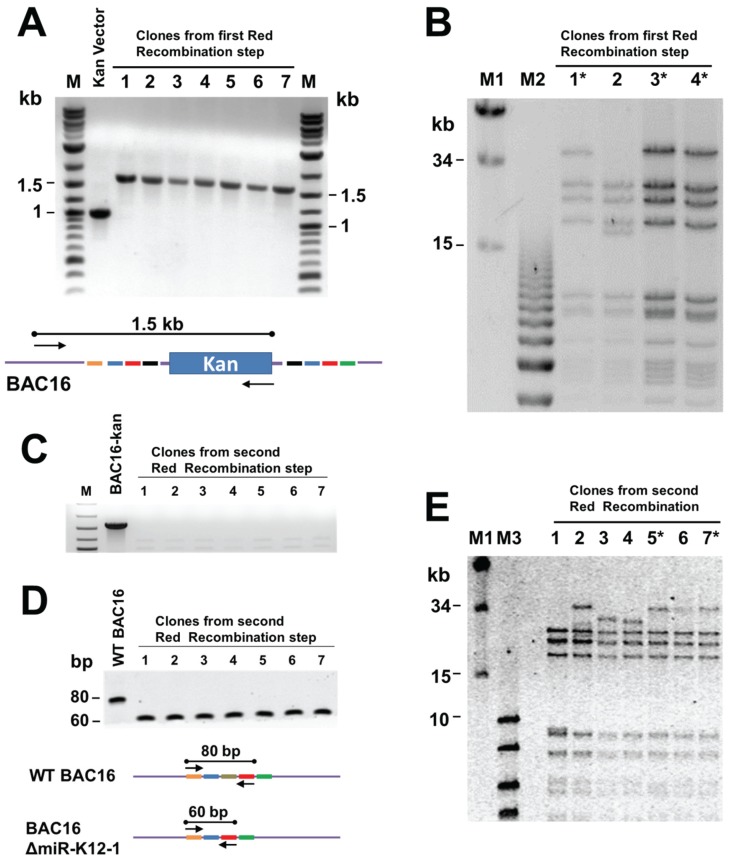
Testing of recombineering products to confirm desired deletion and integrity of terminal repeats. (**A**) PCR products following intermolecular recombination, showing that the kan cassette is present at the intended location. The cartoon below shows primer locations; (**B**) pulsed field gel electrophoresis (PFGE) of *Nhe*I-digested bacmid DNA to verify integrity of TRs in the products of the first recombination step. Clones with the desired *Nhe*I restriction pattern are indicated by asterisks; (**C**) PCR analysis of products from the second (intramolecular) recombination verifying that the kan cassette has been removed. The primers used are represented in the cartoon above; (**D**) confirmation of the deletion by PCR with primers flanking the deleted region, as shown in the diagram below. The wild-type BAC16 gives an 80 bp product and the BAC19ΔmiR-K12-1 mutant a 60 bp product; and (**E**) PFGE to verify TR integrity in the final bacmid recombination products.

Briefly, the kanamycin cassette was PCR amplified using primers with KSHV targeting sequences, and then inserted into BAC16 via Red recombinase in *E. coli* GS1783. The presence of the Kan cassette at the appropriate location in the KSHV genome within BAC16 was confirmed by PCR using one primer against the kan gene and one against KSHV sequences (Figure 3A). Bacmid DNA was isolated from candidate clones and *Nhe*I digests analysed by pulsed field gel electrophoresis to confirm the integrity of terminal repeat sequences (Figure 3B). Bacmids with the correct *Nhe*I restriction pattern were subjected to a second round of Red recombination. At this step the bacmid DNA was linearized by digestion with I-*Sce*I within induced GS1783 cells, and then Red-mediated intramolecular recombination resulted in removal of the kan cassette (Figure 2). Removal of the kan-positive selection marker was verified by kanamycin-sensitivity and by PCR (Figure 3C). The removal of the deleted region was confirmed by PCR, giving a 60 bp product rather than the 80 bp product seen for the wild type genome (Figure 3D). The integrity of the TRs was again tested by PFGE analysis of *Nhe*I restriction digests (Figure 3E).

### 3.3. Generation of iSLK Cells Infected with KSHV miRNA Mutants

**Figure 4:**
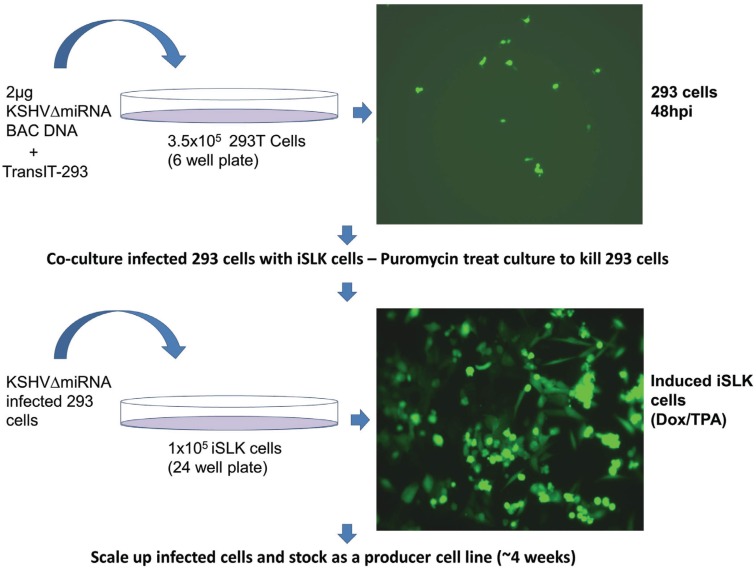
Generation of iSLK producer cell lines by co-culture with bacmid-transfected 293 cells.

Verified bacmids were used to transfect 293T cells. Due to the low infectivity of KSHV it is difficult to amplify the virus using only infected 293T cells. As transfected 293T cells are passaged, the amount of virus produced decreases. Additionally, transfected 293T cells cannot be efficiently reactivated to make a recombinant virus after being thawed from frozen stocks. Instead we used a co-culture system where transfected 293T cells were grown with iSLK cells, which can be readily infected by KSHV. The iSLK cell line was produced [21] by transducing SLK cells [29,30] with an inducible RTA (Replication and Transcriptional Activator) gene, the major KSHV immediate early gene needed for lytic growth. The iSLK cells carry the rtTA (Tet-On) transactivator, which allows doxycycline to activate RTA expression. Infected iSLK cells have been shown to produce much higher titers than 293 cells [21]. Direct transfection of bacmid DNA into iSLK cells was very inefficient, and subsequent infection of iSLK cells with virus derived from transfected 293T cells yielded only low virus titers. To address this issue, we developed a co-culture protocol (Figure 4) which not only increased the efficiency of iSLK infection rate but also yielded much higher titers after iSLK induction with TPA and Valproic acid. The co-cultivation protocol also guarantees that recombinants are replication competent.

### 3.4. TaqMan Analysis of miRNA Mutants Confirms Loss of Expression of Individual miRNAs

**Figure 5:**
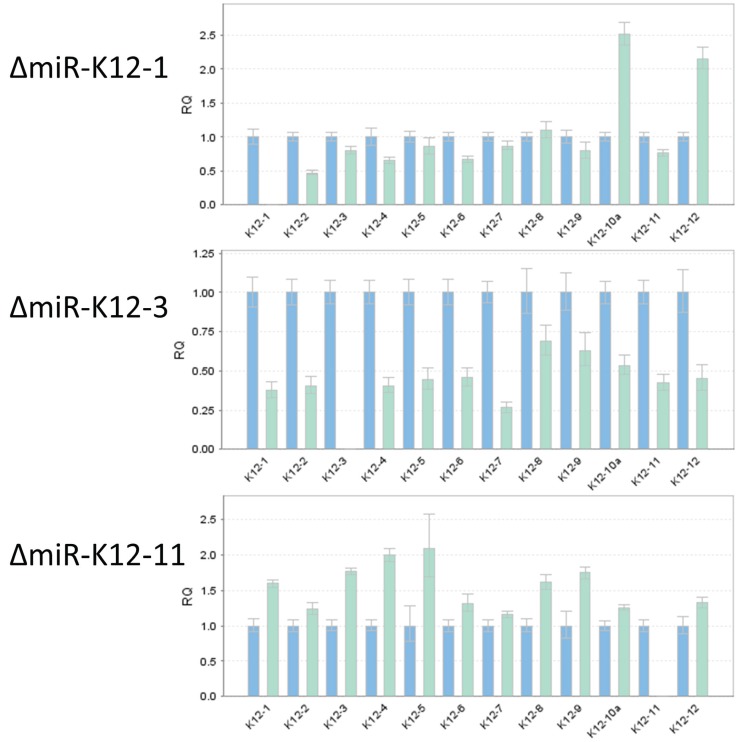
Confirmation of loss of miRNA expression in deletion mutants for miR-K12-1, miR-K12-3, and miR-K12-11. TaqMan miRNA assay was used to determine the levels of mature miRNAs K12-1 through K12-12 (strand-specificity for each Taqman miRNA assay is listed in Table S1). The blue bars show expression for wtKSHV normalized to 1. The cyan bars show expression levels for KSHV deleted for miR-K12-1 (**top**), miR-K12-3 (**center**), or miR-K12-11 (**bottom**).

To confirm that deletion of an individual miRNA resulted in the loss of a specific mature miRNA, we performed TaqMan miRNA assay on RNA independently isolated from individual mutant iSLK virus producer cell lines (Figure 5). As expected, ΔmiR-K12-1 did not show detectable expression of miR-K12-1, and similarly, ΔmiR-K12-3 and ΔmiR-K12-11 lacked expression of miR-K12-3 and ΔmiR-K12-11, respectively. We monitored expression of the remaining miRNAs to ensure that their integrity had not been compromised, and found in all cases that their expression was maintained. We note that the expression levels of neighboring miRNAs did show moderate changes relative to wtKSHV, which is expected since deletion of one hairpin may change pre-miRNA folding and accessibility to Drosha and other cellular factors, which was recently demonstrated by shape analysis of the KSHV pri-miRNA [31]. Additionally, KSHV miRNA expression profiles are cell type specific [32] and even different PEL cell lines show large expression differences [16]. Hence, it will be important to monitor miRNA expression in each newly used cell type. This has already been done for specific KSHV miRNAs that have been studied by utilizing the here-described KSHV mutants [33,34,35].

### 3.5. Illumina Sequencing Shows No Unintended Changes in DNA Sequences of Individual Mutants

We wanted to rule out the possibility that the passage of the bacmid during mutant construction in *E. coli* or during creation of producer cell lines had caused random mutations. DNA isolated from all iSLK producer cell lines was extracted and enriched for KSHV DNA using Agilent SureSelect technology as previously reported [26], and subsequently sequenced by Illumina paired end 55 nt long reads. Whole genome sequencing of “latent” episomes in iSLK producer cell lines, revealed no mutations in wt and mutants in the vast majority of sequence tags. Average coverage per nucleotide was greater than 100 and the few reads that showed a base exchange were single tags suggesting sequencing errors rather than mutations. However, we noted two regions downstream of ORF K9 and miR-K12-9 which had significantly lower coverage (less than 25), possibly due to high GC-rich and repetitive sequences. Importantly, the deletion or mutation for each specific miRNA was confirmed for each producer cell line.

## 4. Conclusions

The objective of this work was to generate a set of quality controlled recombinant viruses that contain defined miRNA mutations on a single genetic backbone of KSHV (BAC16) [20]. This set of mutants, when utilized in a number of laboratories, will also address a major problem in miRNA research in that results often vary when obtained from different strains in addition to different cell systems. To date subsets or the complete set of viral mutants have already been shared with a number of investigators in the US and abroad. As of submission of this manuscript, there have been seven published manuscripts from six laboratories with several manuscripts under submission, which all provide new insights into how KSHV miRNAs contribute to viral biology [33,34,35,36,37,38,39]. While, up to now, most studies on KSHV miRNAs either entailed wt PEL-derived virus or used *in vitro* systems to either inhibit or overexpress miRNAs, these new studies perform the majority of experiments in the context of the viral genome in endothelial cell systems. Hence, our set of mutants has already had some impact and we would hope that they will be useful for many future studies. One challenge however, will be to archive and distribute the recombinants over time since this is associated with considerable cost for maintaining the full set of iSLK producer lines. One way would be to deposit the cell lines in a repository, however at a cost of several hundred dollars per line, obtaining the full set of 15 mutants would be rather expensive. As an alternative, we would hope that laboratories which have obtained our mutants or will obtain them in the future would agree to provide them one time to another laboratory when requested. This way this resource could be maintained and utilized by the KSHV research community for a long time without excessive costs.

## Supplementary Materials

The following are available online at www.mdpi.com/1999-4915/8/2/54/s1, Table S1: KSHV miRNA sequences, Table S2: Primers, Supplementary Protocol 1: Bacmid mutagenesis, Supplementary Protocol 2: Generation of virus producer cell lines.
