# Supplementary material for: A Toolbox for Herpesvirus miRNA Research: Construction of a Complete Set of KSHV miRNA Deletion Mutants
Source: Viruses. 2016 Feb 19;8(2):54. doi: 10.3390/v8020054 (PMC4776209; doi:10.3390/v8020054)
Supplement: Supplementary file 1 [file viruses-08-00054-s001.zip › viruses-113147-supplementary (final)/Supplementary Protocol 1.pdf]

Supplementary Materials: A Toolbox for Herpesvirus miRNA Research: Construction of a Complete Set of KSHV miRNA Deletion Mutants

Vaibhav Jain, Karlie Plaisance-Bonstaff, Rajnikumar Sangani, Curtis Lanier, Alexander Dolce, Jianhong Hu, Kevin Brulois, Irina Haecker, Peter Turner, Rolf Renne and Brian Krueger

Supplementary Protocol 1: Bacmid Mutagenesis

Outline:

Day 0
Inoculate medium with *E. coli* GS1783 containing BAC16
Day 1
Prepare fresh GS1783/BAC16 competent cells.
PCR amplify Kan^R targeting construct, *Dpn*I digest, gel purify and electroporate
Day 2
Colony PCR colonies recovered from Kan plate and inoculate for miniprep
Design restriction enzyme digestions to identify correct clones
Analyze results from colony PCR
Day 3
Mini prep, restriction enzyme (RE) digestions, and gel electrophoresis (o/n PFGE)
Day 4
Analyze results from RE digestions
Inoculate for second recombination
Day 5
Perform I-*Sce*I induction and 2nd Red recombination to eliminate Kan marker
Plate bacteria on Cm ara.
Day 6
Replica plating of clones onto Cm and Kan plates
Day 7
Identify Cm resistant/Kan sensitive clones
Colony PCR and inoculate for miniprep
Design RE digestions
Analyze results from colony PCR
Day 8
Miniprep and digest clones
Day 9
Analyze results of RE digestion

References

1. Tischer, B.K.; von Einem, J.; Kaufer, B.; Osterrieder, N. Two-step red-mediated recombination for versatile high-efficiency markerless DNA manipulation in *E. coli. Biotechniques* **2006**, *40*, 191–197.
2. Tischer, B.K.; Smith, G.A.; Osterrieder, N. En passant mutagenesis: A two step markerless red recombination system. *Methods Mol. Biol.* **2010**, *634*, 421–430.
3. Brulois, K.F.; Chang, H.; Lee, A.S.; Ensser, A.; Wong, L.Y.; Toth, Z.; Lee, S.H.; Lee, H.R.; Myoung, J.; Ganem, D.; *et al*. Construction and manipulation of a new Kaposi's sarcoma-associated herpesvirus bacterial artificial chromosome clone. *J. Virol.* **2012**, *86*, 9708–9720.

BACmid Mutation Primer Generation Protocol (adapted from Tischer *et al.*, 2006)

Designing for Deletions

Determine the sequence to be deleted.
For the forward primer, select 40 bases upstream of the site to be deleted and 20 bases downstream of the site to be deleted to make a 60 bp primer and then add on the positive selection marker (PSM) forward sequence (See Figure 1). F-AGGATGACGACGATAAGTAGGG
R-AACCAATTAACCAATTCTGATTAG
For the reverse primer, select 40 bases downstream of the mutation site, 20 bases upstream of the mutation site, and generate the reverse complement. Add on the positive selection marker reverse sequence (See Figure 1).

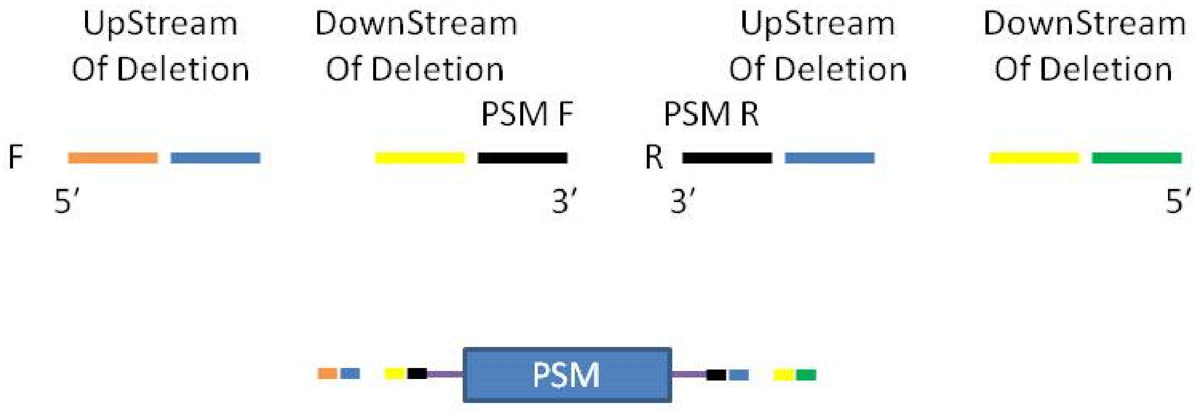

Figure 1. Assembling a cassette with the positive selection marker (PSM, kanamycin-resistance) flanked by copies of the deleted region.

Designing for Insertions

30 bp of DNA can be reliably inserted using this method.
The primer design is the same as for the deletions, except the sequence to be inserted is added in between the upstream and downstream sequences in the primers. (See Figure 2)

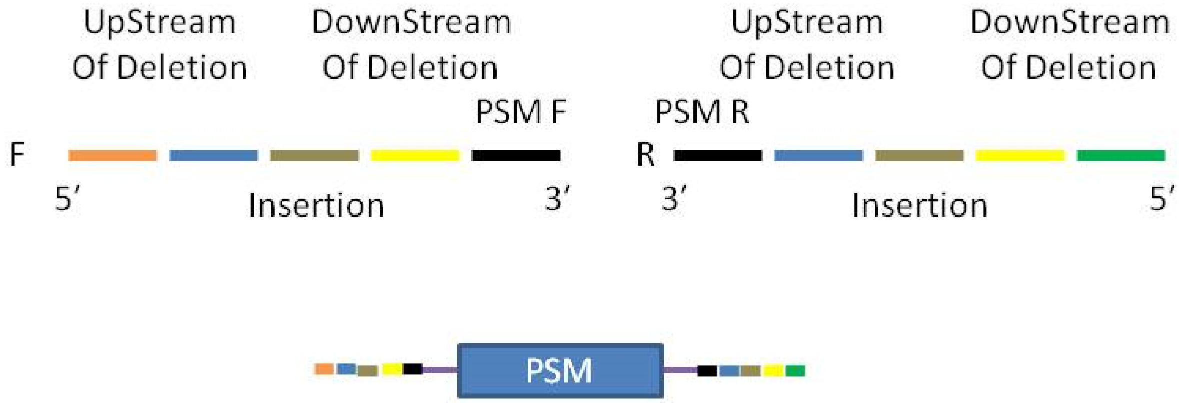

Figure 2. Assembling a cassette with the positive selection marker in between copies of the inserted region.

Technique Overview

Generation of Red-Recombination Cassettes

Materials:
Plasmid pEP-KanS
Phusion High Fidelity Taq

IDT ultramer Primers, PAGE-purified (100 μM)

PCR Reaction and Conditions

Set up a standard (Follow Phusion instructions) 150 μL reaction for each primer set
(0.3 μM~10 pM/μL of each primer)
Use only **1 ng** of targeting construct as the template
Cycling conditions are standard (follow Phusion instructions), except only run for 20 cycles

PCR Cycle Conditions:

Temp. (°C)	Time
98 °C	1 min
98 °C	30 s
55 °C	30 s
72 °C	60 s
Repeat from step 2:	20×
72 °C	300 s
4–8°C	forever

Aliquot into 50 μL reactions before loading into the cycler
After run, pool the PCR reactions and add 3 μL of *Dpn*I restriction enzyme to the pooled PCR reactions
Incubate in the heat block at 37 °C for 2 h
During the incubation, make a 1% agarose-TBE gel
Tape lanes on the comb together (3) to make wells large enough to hold 150 μL of digested DNA
Add 5× loading dye to the digestions
Run on the gel
Verify size of PCR product (1.5 kb) and then gel purify the fragment using Qiagen Gel Extraction Kit
Resuspend gel purified DNA in 40 μL of H_2O

Gel Purification of Kan Cassette

The PCR product was purified following electrophoresis on a 0.8% agarose-TAE gel using the QIAEX II Gel Extraction kit (Qiagen), following the manufacturer's protocol. DNA was eluted into 20 µl of 10mM Tris-Cl, pH 8.5.

Generation of Electrocompetent GS1783 *E. coli*

Materials:
WT GS1783 cells containing KSHV BAC16
Shaking incubator set to 30 °C
Water bath set to 42 °C
LB-Cm plates (15 μg/mL Chloramphenicol)
Centrifuge set to 0 °C
Ice cold sterile water
Ice cold 10 mL Pipettes
Ice cold 15 mL culture tubes

Overnight Culture

Grow an overnight culture (30 °C, 220 rpm) of GS1783 in 25 mL LB Cm in a 50 mL conical tube (Tape the cap on loosely).

Morning Culture

Inoculate 40 mL of LB Cm in a sterile 250 mL flask with 2 mL of the overnight culture
Shake at 220 rpm and 30 °C until the culture reaches an OD600 of 0.5 (~2 h)

Induce Red Expression

Transfer the culture to a 42 °C waterbath and then shake vigorously by hand for 10 min to ensure rapid heating of the media to 42 °C to induce Red expression
Place the culture in a wet ice slurry and chill for at least 10 min.
Set centrifuge chilling to 0 °C

Make Competent Cells by Washing in Water

Aliquot the 40 mL culture into 4 × 15 mL culture tubes and spin at 3000 g for 8 min
Wash 3× with sterile ice cold ddH_2O (The pellet will become very loose, so be careful when removing the supernatant).
After the last wash, resuspend the pellets in the residual H_2O (approx. 200 µL) and combine into a single prechilled Eppendorf tube
Continue on to electroporation of the DNA cassette

Electroporation of Cassettes for Red Recombination

Materials:

BioRad GenePulser Xcell
LB-Kan plates (20 µg/mL Kanamycin)
Freshly made/digested/gel purified PCR product of the cassette
Freshly made electro-competent cells

Electroporation

Combine 10 µL (~100 ng) of your purified DNA with 40 µL of electrocompetent cells in a chilled Eppendorf tube
Flick tubes to mix the cells and DNA
Transfer to a pre-chilled electroporation cuvette
Flick cuvette to be sure there are no bubbles between the cuvette electrodes
Verify that the liquid level on each side of the cuvette is the same
Turn on the GenePulser
The parameters for the BAC electroporation are 1.5 kV (1500 V), 25 µF, 200 ohms, and 1 mm cuvette
Once the parameters are verified, insert the cuvette into the shock pod.
Press the pulse button
Verify that the time constant is between 3.5 and 4.5 ms

Recovery

Add 250 µL of SOC media to the cuvette
Mix and transfer to an Eppendorf tube
Incubate the electroporated *E. coli* at 30 °C and 220 rpm for 1 h
Plate 200 µL on LB-Kan plates and grow at 30 °C for 24 h

Verify

Pick 8 colonies and perform colony PCR with the appropriate forward primer and Kan reverse primer, start mini-cultures for BAC digestion and streak on Kan plates.
Verify the insertion by colony PCR.
Verify that the terminal repeats are intact by *Nhe*I digestion and pulsed field gel electrophoresis.

Continue with clone(s) that satisfy the verification criteria.

Minipreps of BAC clones and low copy plasmids

1. Grow cells o/n in 5 (15 mL tube)–10 (50 mL tube) ml LB with antibiotics.

Optional: Make 500 µl glycerol stock

2. Centrifuge culture at 4000 rpm 10 min
3. Discard supernatant and **completely** resuspend cells in 300 µL Qiagen P1 or similar (e.g., 50 mM glucose, 25 mM Tris-HCl, pH 8, 10 mM EDTA, 100 µg/mL RNase). Transfer to a 1.5 mL Eppendorf
4. Add 300 µL **P2** (Qiagen, 0.2 M NaOH, 1 % SDS)—Mix by inverting until CLEAR (you can use the Qiagen blue dye to verify). **DO NOT VORTEX to avoid shearing.** Leave **MAX. 5 min** at room temperature
5. Add 300 µL **P3** (Qiagen, 5 M K acetate: make by mixing 60 mL 5 M K acetate, 11.5 mL glacial acetic acid, and 28.5 mL water). Mix completely by inverting. **DO NOT VORTEX to avoid shearing. Also avoids contamination with *E. coli* genomic DNA.**

Optional: put on ice for 10 min

6. Centrifuge at 13,000 rpm at RT or 4 °C for 10 min
7. Transfer clear supernatant to a 2 mL Eppendorf (If supernatant is not clear, pour in another tube and repeat step 6.
8. Add 600 µL **isopropanol** and mix well by inverting. Centrifuge at 13,000 rpm at RT or 4 °C for 30 min.
9. Pipet off supernatant; be careful not to remove the pellet. Small pellet: avoid using vacuum suction.
10. Add 1 mL **70% EtOH** (made with pure (dd) water), invert a few times to wash. Centrifuge at 13,000 rpm at RT for 10 min
11. Carefully remove all traces of ethanol without touching the pellet, then **Air dry the pellet**
12. Resuspend carefully in 20–50 µL TE (by waiting and a few inversions; do not vortex) (use water here when immediate sequencing is intended: EDTA compromises sequencing reactions)

This DNA contains residual nucleotides. OD260 will overestimate!

Determine DNA quantity by Gel electrophoresis.

CHEF Pulsed Field Gel Electrophoresis (PFGE) Protocol

Materials

CHEF Pulsed Field apparatus (BioRad CHEF-DR II).
3 L 0.5× TBE
NEB Megabase ladder N3551S
Certified Megabase Agarose (BioRad, cat # 161-3108)
Gel casting apparatus

Chill Buffer

Fill gel tray with 3 Liters of 0.5× TBE running buffer
Turn on the circulation pump
Run the pump until all of the bubbles are blown out of the system
Plug in the Chiller and set the temperature to 47 F
Run the Chiller for 30 min
During this time pour the gel

Pouring the Gel

Add the black tray to the gel casting box
Tape the outer side of the gel box with 1″ masking tape and make sure there's a tight seal
Make a 150 mL 1% gel with pulsed field certified megabase agarose in 0.5× TBE
Pour 10 mL of this agarose into a 50 mL beaker and save for later
Pour the gel in the gel tray
Balance the gel comb on two eppendorf trays (Lower lip of the gel tray)
Let the gel solidify
Once solid, remove the comb and add the megabase ladder
Cut a thin slice of megabase ladder from the agarose plug using a razor
Slide the gel slice into the gel well at each end of the gel
Re-heat the 10 mL of saved gel from earlier
Cool for 10 min
Pipette the gel into the wells with the megabase ladder without making air bubbles
Once solid, remove the excess gel with your finger
Place into the cooling gel buffer
Let the gel cool for 15 min
Prior to loading, turn off the chiller by unplugging it (Be sure the compressor isn't running before unplugging! DO NOT UNPLUG IF THE GREEN LIGHT IS ON!!)
Let the pump run for 5 min to prevent buffer from freezing in the chiller
Turn off the pump
Load the gel

Single Power Supply Pulse Field Protocol (CHEF-DR II System)

Turn on the controller (switch in back) and power supply (switch on side)
Press the block button and set to 1
Set the initial time to 1 and the final time to 1
Set the run time to 0.6 (40 min)
Set the volts per channel to the max (6)
Change the block to 2
Set the initial time to 1 and the final time to 5
Set the run time to 16 h
Set the volts per channel to the max (6)
Push Start

Let the run go for 40 min and then check to be sure the DNA has entered the gel
DO NOT FORGET TO CHECK THE GEL, IT WILL MELT IF YOU LET IT RUN WITHOUT THE CHILLER
Turn on the pump
Plug in the chiller

Second Red Recombination and Verification of PSM Removal

Materials:

20% arabinose (Ara)
Cm/Ara round plates (15 μg/mL Cm, 1% Ara)
Cm/Ara replica plates (15 μg/mL Cm, 1% Ara)
Kan/Ara replica plates (20 μg/mL Kan, 1% Ara)
LB-Kan plates (20 μg/mL Kan)
LB-Cm plates (15 μg/mL Cm)

Overnight Culture

Once the insertion is verified by colony PCR and the KSHV terminal repeats are determined to be intact, inoculate an overnight culture with the verified clone (25 mL LB Kan in a 50 mL conical, 30 °C, 220 rpm)

Morning Culture

Inoculate 40 mL of fresh LB Cm (**No kanamycin, the Kan resistance is being removed**) with 2 mL of the overnight culture in a sterile 250 mL flask.
Grow to an OD_{600} of 0.5.

Induction of I-*Sce*I and Red

Induce I-*Sce*I expression by adding Arabinose to a final concentration of 1% (4 mL of a 20% stock)
Shake for 45 min at 30 °C
Induce Red expression by incubating the flask with vigorous shaking in the 42 °C waterbath for 10 min
Shake again at 30 °C for 2 h
Make a 10-fold serial dilution series in 1.5 mL Eppendorf tubes (100 µL into 900 µL media, $10^{-1, -2, -3 \ldots -9}$)
Plate 100 µL of the original stock, -3, -5, -7, and -9 dilutions on Cm/Ara plates and grow for 24 h

Verify Recombination

Plate single colonies on Cm/Ara and Kan/Ara replica plates to identify Cm resistant but Kan sensitive colonies
Verify that terminal repeats are intact by PFGE.
Verify Kan loss using verification primers to upstream and downstream mutation regions
If deletion, verify length of region by TBE Urea PAGE (6% Acrylamide). Should be reduced relative to the size of the wild type PCR product by the size of the deletion.

© 2016 by the authors; licensee MDPI, Basel, Switzerland. This article is an open access article distributed under the terms and conditions of the Creative Commons by Attribution (CC-BY) license (http://creativecommons.org/licenses/by/4.0/).
