# Supplementary material for: A Toolbox for Herpesvirus miRNA Research: Construction of a Complete Set of KSHV miRNA Deletion Mutants
Source: Viruses. 2016 Feb 19;8(2):54. doi: 10.3390/v8020054 (PMC4776209; doi:10.3390/v8020054)
Supplement: Supplementary file 1 [file viruses-08-00054-s001.zip › viruses-113147-supplementary (final)/Supplementary Protocol 2.pdf]

Supplementary Materials: A Toolbox for Herpesvirus miRNA Research: Construction of a Complete Set of KSHV miRNA Deletion Mutants

Vaibhav Jain, Karlie Plaisance-Bonstaff, Rajnikumar Sangani, Curtis Lanier, Alexander Dolce, Jianhong Hu, Kevin Brulois, Irina Haecker, Peter Turner, Rolf Renne and Brian Krueger

Supplementary Protocol 2: Generation of iSLK virus producer cell lines by co-culturing transfected 293T cells with iSLK cells

Outline:

A. 293T Transfection (2–3 weeks)
B. Co-culture to create infected iSLK virus producer cell line (2–3 weeks)
C. Induction of virus production in iSLK Cells (1–2 weeks)
D. Freezing and thawing cells
E. Virus collection from iSLK producer cell lines
F. KSHV DNA isolation from cell cultures
G. qPCR Virus Stock DNA for Copy Number
H. Qiagen Large-Construct DNA Isolation * or
I. BAC miniprep for transfection grade DNA *
* Either H or I can be used for isolation of bacmid DNA

References and Notes

1. Myoung, J.; Ganem, D. Generation of a doxycycline-inducible KSHV producer cell line of endothelial origin: Maintenance of tight latency with efficient reactivation upon induction. *J. Virol. Methods* **2011**, *174*, 12–21.
2. Brulois, K.F.; Chang, H.; Lee, A.S.; Ensser, A.; Wong, L.Y.; Toth, Z.; Lee, S.H.; Lee, H.R.; Myoung, J.; Ganem, D.; *et al*. Construction and manipulation of a new Kaposi's sarcoma-associated herpesvirus bacterial artificial chromosome clone. *J. Virol.* **2012**, *86*, 9708-9720.
3. Mirus TransIT®-293 Transfection Reagent Protocol http://www.mirusbio.com/assets/protocols/ml009_transit_293_transfection_reagent.pdf (accessed on 17 February 2016).
4. Qiagen large construct kit (cat # 12462) and Bac miniprep kit

Important: all steps described in this protocol should be performed under P2* conditions by laboratory personnel who have received appropriate training.

Materials

Mirus TransIT©-293 Transfection Reagent (Mirus Cat No. MIR 2700).
Serum Free Media, Complete DMEM, BACmid DNA, Sodium butyrate, 10 mg/mL Doxycyline stock, 25% Sucrose filter sterilized, TPA, valproic acid, 10 cm plates, 15 cm Plates, 6-well plates, Beckman Polyallomer Tube for SW28 rotor. [cat no. 326823], SW28 rotor for virus collection.

Complete DMEM

To Cellgro 1X DMEM (cat # 10-017-CM) add. fetal bovine serum (FBS) to 10% final conc.
100 units/mL penicillin G-final conc.
100 μg/mL streptomycin-final conc.

For Infected 293T Cells: Make Fresh

Add Hygromycin to final conc. 100 μg/mL in complete DMEM

293T Induction Media (Induction of Viral Replication) 25 mL. Make Fresh!
A. 25 mL complete DMEM**. No antibiotics**
B. TPA : final concentration 20 ng/mL
C. valproic acid: final conc 1 μM.

Infected iSLK Media: Make fresh

25 mL complete DMEM,
1 μg/mL Puromycin (can vary up to 10 μg/mL final conc.)
250 μg/mL G418-final conc.
1200 μg/mL Hygromycin—final concentration

iSLK Induction Media (Induction of Viral Replication) 25 mL: Make Fresh!
A. 25 mL complete DMEM
B. 2.5 μL of a 10 μg/μL stock solution of doxycycline for a final concentration of 1 μg/mL
C. Sodium butyrate for a final concentration of 1 mM

Freezing Media—3 mL

50% FBS (1.5 mL)**,** 10% DMSO (300 μL), 40% DMEM (1.2 mL)

A. 293T Transfection (2–3 weeks)

Note: The DNA for transfecting 293T cells should be isolated using the large construct kit protocol as in section H (Qiagen). This kit has an exonuclease digestion step, which is crucial for DNA purity. You can add this step if you use some other BAC isolation protocol. This transfection protocol is adapted and modified from Mirus Transfection reagent protocol. Please refer to Mirus website (https://www.mirusbio.com/products/transfection/transit-293-transfection-reagent) for technical details. Approximately 24 h prior to transfection, plate 293T cells in 6 well plate at a density of 1 × 10^5 cells/well in a total volume of 2 mL DMEM in each well (6 wells/mutant or Bac prep). Run WT BAC control experiments in parallel.

1. Incubate cells overnight at 37 °C in a CO_2 incubator
2. The 293T cells should be 50%–70% confluent before transfecting
3. In a sterile microcentrifuge tube add the Mirus TransIT©293 reagent (3 μL per well for 6 well plate wells) directly into 200 μL of serum-free DMEM media—for 6 wells add 18 μL of Mirus Trans IT©-293 Reagent in 1200 μL of serum free media
4. Mix gently by inverting the tube few times.
5. Incubate at room temperature for 20 min.

6. Add 2 µg DNA/well to 293 Mirus Trans IT©-293 Reagent and mix by gentle pipetting (6 µg total DNA for 3 wells/Bac DNA).
7. Incubate at room temperature for 30 min.
8. Add 200 µL of the Trans IT©-293 Reagent/DNA complex mixture prepared in steps 3–7 dropwise to each well containing cells in the 6 well plate.
9. Gently rock the dish back and forth and from side to side to distribute the complexes evenly (Do not swirl the plate).
10. Incubate for 24 h observing GFP expression in cells. Continue observing cells, keep changing media as needed. This really depends on cell death and the color of the media. Add Hygromycin, when proportion of GFP producing cells reaches about 10%. You should see a lot of cell death after adding selection media. Non transfected cells tend to dislodge transfected cells with them, but if you have some GFP cells still attached to the well, replace the media with fresh DMEM and add the antibiotics the next day. If you don't see a lot of GFP positive cells attached to the plate, then remove the media with dead cells, centrifuge (1500 RPM for 8 min. in bioshield-1000 rotor) it and re-suspend gently but thoroughly in DMEM (no antibiotics) and seed it in a 10 cm plate. Watch for GFP+ cell reattachment and add antibiotics the next day. Do not discard your 6 well plate and keep changing the media in it. Left over cells in 6 well plates grow in about 2 weeks.
11. Harvest cells at 50%–80% confluency of GFP producing cells in 6 well plate, and transfer into 10 cm plate-you can transfer all 3 wells into one 10 cm plate.
a. In order to harvest the cells from 6 well plates, add 500 µL trypsin/well allowing cells to detach completely from plate then add 1.5 mL of complete DMEM media. Mix the cells well before transferring.
b. Remove total cell culture content of 6 mL (triplicate) from 3 wells and transfer to a 10 cm plate.
12. Next pipette 4 mL of fresh complete media for a 10 mL cell culture total volume into 10 cm plate. Incubate overnight and next day add Hygromycin to 100 µg/mL (20 µL Hygromycin 50 mg/mL stock per 10 mL of media).
13. Continue to maintain 293T cell culture until 70%–80% cell confluency. If you need more cells at this point, you can expand the cells by splitting them appropriately.
14. Maintain the 293T cells by splitting them 1:10 in infected 293T media every two days. You can freeze them for future experimentation
15. You will need a total of approx. 750,000 of each cell type (iSLK & 293T) to start co-culture in one 6 well plate per mutant.

B. Co-culture experiment for creating iSLK virus producer cell line (2–3 weeks)

16. Thaw iSLK cells and keep maintaining them in uninfected iSLK media by splitting them 1:10 every two days. You will need them in step 18 below
17. In a new 6 well plate, seed approximately 1.25×10^5 ransfected 293T cells per well in infected 293T media (cDMEM + Hygro). Always run WT BAC as control in a separate plate
18. Next day induce the cells for the production of virus by replacing the media with 293T induction media. Incubate in 37 °C CO2 incubator for 48–72 h
19. After induction **transfer** the used media in a Falcon tube *(see note below)*, **trypsinize** the cells in the 6 well plate and count the cells. Also trypsinize and count the iSLK cells. You will need approx. 1.25×10^5 each cell type per well
20. Mix iSLK: 293T cells in 1:1 ratio (total 250,000 each cell per 2 mL complete DMEM or used induction media/ well-No antibiotics) in 6 well plate incubating at 37 °C CO_2 incubator.

Note: *Here I divide the plate in equal halves and I use the old induction media collected in the falcon tube above in the top three wells and in bottom 3 wells, just add fresh media* ***without antibiotics****. There can be various arguments for keeping the induction media while adding iSLK cells, but in some cases if the infectivity is low, keeping the induction media on is the only way to get efficient virus infection rate. In*

the ideal situation, if everything goes well, you should see the infections in each well and at that time you can simply pick the most suitable wells for your future experiments.

Since each mutant is different, the steps following these steps are based on my experience with most mutants. But induction time, addition of hygromycin and selection of iSLK cells vary for each experiment. Given the 6 wells, I vary these conditions as I go along and apply what is needed to see the infected cells. It is therefore advised to keep watching the cells for infected iSLK cells, overgrowth etc. and change media at the appropriate time.

21. In the top 3 wells where you added the used induction media, change the media 2 day post mixing the cells. In the bottom 3 wells you can change the media 2–4 day post mixing.

22. After 2–4 days, if the cells are getting overcrowded, replace the media (2 mL) with fresh media for infected iSLK (final antibiotic concentrations: 1 μg/mL Puromycin, 250 μg/mL G418, and 1200 μg/mL Hygromycin). Keep incubating the cells in this media for selecting infected iSLK cells.

Again, you can first select for iSLK cells alone by not adding hygromycin in some wells. iSLK cells have endogenous G418 and Puromycin resistance markers.

23. Wash cells with PBS or media when needed (avoid back to back washes) and repeat step 19 when necessary to remove dying 293T & uninfected iSLK cells. You should start seeing patches of GFP+ iSLK cells about 1 week post infection.

24. Once confluency of GFP^+ iSLK cells reaches 70%–90% in 6 well plate, and unwanted cells have been removed by selection, transfer cells by combining all equivalent wells to one 10 cm plate. I make two separate plates if I induced them with different conditions. Avoid adding selection immediately after transfer. Add selection after overnight incubation.

25. At about 80%–90% confluency, transfer the cells into a 15cm plate with infected iSLK media. Freeze the cells before further experimentation.

C. Induction of Virus Production in iSLK Cells

26. Once 10 cm plate is 70%–80% confluent move to 15 cm plate (2–4 days, depends upon initial confluency).

(Based on your requirement, you can use one 15 cm plate or triplicate. I always use triplicate). Do not forget to keep some cells growing in 10 cm plate for future use. **Also grow enough to freeze cells for future use.**

27. Grow infected iSLK cells in triplicates of 15 cm plates in infected iSLK media.

Follow freezing and thawing protocol. To ensure that virus production is not affected by freezing and thawing, you can also use thawed & regrown healthy cells for inducing virus production & quantification.

28. When cells are 50%–60% confluent, transfer into triplicates of 15 cm plate. Induce with 1 μg/mL of DOX and 1 mM of NaB per plate or you can use same induction media mentioned above.

29. 4 days post induction collect virus by following virus collection protocol below in **section E.**

30. Purify virus DNA from collected virus for qPCR quantification.

D. Freezing and Thawing Cells

31. Freeze cells by transferring 1×10^6 cells per 1 mL freezing media into designated cryovials and follow slow freezing steps.

32. Follow quick thawing protocol at 37 °C

33. After overnight incubation add 1 μL of Puromycin (stock of 10 mg/mL) and 50 μL G418 (stock 50 mg/mL) to 10 mL medium, with appropriate selection for BACmid.

E. Virus Purification from iSLK Cells Four Days After Induction

- **Virus culture filtration**
- Four days after induction, draw cell culture supernatant into a 30 mL syringe and pass through a 0.45 micron syringe filter into a 50 mL tube.
- Discard filter into bleach waste container. Draw bleach into the syringe and dispense onto the plate of cells to disinfect.
- The supernatant may be stored at 4 °C up to 5 days prior to ultracentrifugation.

Ultracentrifugation

- Spray centrifuge rotor cups with 70% ethanol inside hood.
- Leave in hood under UV light for 10–15 min.
- Pipet 5 mL of a 25% sucrose solution into a polyallomer (Beckman cat# 326823) centrifuge tube.
- Carefully add the virus-containing supernatant drop-wise to the center of the sucrose solution, making sure that mixing does not occur. The sucrose solution and the supernatant should form 2 distinct layers.
- Add serum-free DMEM to the centrifuge tube in order to fill it completely (approximately 35 mL). This will prevent the tube from collapsing during centrifugation.
- Centrifuge for 1 h at 25,000 rpm and 4 °C.
- Remove tube from rotor cup and check for leakage. If leakage has occurred, immediately disinfect the rotor cup with 70% ethanol, bleach and expose to UV light once virus collection procedure is completed.
- Remove medium by carefully tilting tube and aspirating, making sure not to disturb the virus pellet.
- Allow pellet to air dry for 2–3 min.
- Add 200 μL of serum-free DMEM (approximately 0.7% of the original sample volume).
- Vortex gently for 15 s.
- The virus stock may be stored at 4 °C for up to 3 days.

F. DNA Isolation

- Pipet 25 μL of virus stock into a 2 mL tube with a conical bottom. Add 250 μL DNAzole and mix by pipetting.
- Centrifuge 10 min at 10,000× *g* and 4 °C or room temperature. Transfer the resulting viscous supernatant to a fresh tube.
- Add 125 μL 100% ethanol and 1 μL 20 mg/mL glycogen (5 Prime).
- Invert tube several times and let stand on ice 5 min.
- Centrifuge for 30 min at top speed (approximately 16,000× *g*) and 4 °C.
- Carefully remove the supernatant by tilting the tube in the direction opposite of where the pellet should be (it will probably not be visible). Hold the tube at this angle for approximately 30 s, until all of the ethanol has run down the wall of the tube and collected at the mouth. Aspirate off the remaining ethanol and, keeping the tube at the same angle, air dry pellet in hood for 15 min.
- Resuspend pellet in 25 μL autoclaved ddH_2O.
- Store DNA at −20 °C.

Virus quantification

- Use 1 μL of viral DNA per qPCR reaction.
- Calculate the number of viral genome copies per mL. For ex. if 30 mL of virus-containing supernatant was concentrated into 200 μL of virus stock. 30,000 μL ÷ 200 μL = 150, making this

a 150-fold enrichment. 1000 μL ÷ 150 = 6.6. To obtain viral genome copies per mL, multiply the number of PCR genome copies per μL by 6.6.

G. <u>qPCR Virus Stock DNA for Copy Number</u>

1. Thaw all reagents completely, quick spin, vortex, quick spin again, keep on ice
a. Virus DNA
b. Standard DNA (pcDNA3.1 ORF73)
c. Primers (LANA N-terminus)
d. SYBR master mix (ABI FAST SYBR-20)
e. qPCR water (autoclaved ddH2O, only open in hood, not at bench)
2. Design plate layout and calculate master mix
a. Each well receives 10 μL SYBR, 1 μL primer, 8 μL ddH2O, and 1 μL DNA
b. I usually calculate so that I can make enough for a master mix for each triplicate (making enough for 4 wells for pipetting error) Also make enough for water controls
c. Example 4 DNA samples (2 dilutions for each = 12) + 5 for standard = 17 × 4 = 68 + 4 (for 1 water control and 3 for error) = 72 so 720 μL sybr, 72 μL primer, 576 μL water
3. Do all work in PCR hood. Label 1.5 mL tubes (1 tube for each sample)
4. Mix SYBR, primers, and water. Pipette 76 μL into each 1.5 mL tube
5. Take primers out of hood and bring in virus DNA.
6. Make x2 ten-fold dilutions of each virus DNA
7. Add 4 μL of DNA to each corresponding 1.5 mL master mix tube
8. Take virus DNA out of hood and bring in standard DNA
9. Make a 5 point ten-fold dilution series for the standard.
a. 5 ng through 0.5 pg (equals to 5.6×10^8 through 5.6×10^4 molecules)
b. I usually make these dilutions fresh for each PCR, if you want to make a stock of each dilution make sure you use low adhesion tubes
10. Add 4 μL of standard DNA to corresponding 1.5 mL master mix tube
11. Vortex all master mix tubes and quick spin.
12. Pipette 3 wells for each master mix tube onto ABI 48/96 plate
13. Cover plate with film
14. Spin plate down (1000 rpm for 1 min)
15. Load plate into ABI StepOne qPCR machine
16. Open Step One software
17. Click advanced setup
18. Enter experiment name
19. Choose which machine you are using (48 *vs.* 96 well)
a. Quantitation (standard curve)
b. SYBR green reagents
c. Fast run (40 min)
20. Click plate setup
a. Enter primer names under target and DNA names under samples
b. Click assign targets and samples
c. Define and setup standards
i. # of points = 5
ii. # of replicates = 3
iii. Starting quantity = 560000000
iv. Serial factor = 1:10
v. Choose wells of standard then close
d. Highlight wells and check primer (target) and DNA (sample)
e. For water control highlight well, check primer then gray N next to primer for negative control

f. Check that Run method is correct
i. 95 for 20 s
ii. 95 for 3 s
iii. 60 for 30 s
iv. 95 for 15 s
v. 60 for 1 min
vi. 95 for 15 s

g. Click Run
i. Choose where to save your data
ii. Wait and make sure that the machine moves the plate up to the heat block and that your run starts (it will show how much time is left)

H. LARGE-CONSTRUCT DNA ISOLATION - Qiagen

Important notes before starting:

- Add RNase A to P1 if not already in use (one vial of RNase A per bottle of Buffer P1 for final concentration 100 µg/mL)
- Prechill Buffer P3 to 4 °C
- Prewarm elution Buffer QF to 65 °C

Protocol:

1. Isolate a single colony from a freshly streaked selective plate and inoculate into 2–5 mL LB medium containing antibiotic for 8 h at 30 °C at 300 rpm.
2. Dilute 0.5–1.0 mL of culture into 500 mL selective LB medium and grow at 30 °C for 12–24 h at 300 rpm.
3. Centrifuge at 6000× *g* for 10−15 min at 4 °C.
4. Resuspend the bacterial pellet in 20 mL Buffer P1 by vortex (40 mL for 1 L Prep, keep in 250 mL bottle!!).
5. Add 20 mL Buffer P2, mix gently (do not vortex) by inverting 4–6 times, and incubate at room temperature for 5 min (40 mL for 1 L prep).
6. Add 20 mL chilled Buffer P3, mix immediately but gently by inverting 4–6 times, and incubate on ice for 10 min (40 mL for 1 L Prep).
7. Centrifuge at ≥20,000× *g* for 30 min at 4 °C and remove supernatant promptly when complete (alternative is to centrifuge at 6000 rpm for 15 min at 4 °C in 50 mL Falcon tube)
8. Pre-wet filter paper with deionized water
9. Filter the lysate through a folded wet filter (fold the filter paper into a cone shape in order to pour the supernatant slowly for filtering into 50 mL Falcon tube)
10. Precipitate DNA by adding 36 mL (0.6 volumes) room temperature isopropanol to the cleared lysate. Mix and centrifuge immediately at ≥15,000× *g* for 30 min at 4 °C. (alternatively is to centrifuge at 6000 rpm for 45 min at 4 °C in 50 mL Falcon Tube) [72 mL for 1 L prep].
11. Resuspend the exonuclease contained in each vial in 225 µL Exonuclease Solvent before use. Mix by tapping the vial, and leave standing for 15 min to allow complete solution of the freeze-dried enzyme. **Do not Vortex.**
12. Carefully decant the supernatant without disturbing the pellet.
13. Wash DNA pellet with 5 mL room temperature 70% ethanol, and centrifuge at ≥15,000× *g* for 15 min. Carefully decant the supernatant without disturbing the pellet (alternative is to centrifuge at 6000 rpm for 15 min at 4 °C in 50 mL Falcon tube). [10 mL for 1 L Prep].
14. Place the tube containing the DNA pellet upside down on a paper towel and allow the DNA to air dry for 2–3 min. Carefully remove any additional liquid visible on the tube opening and carefully redissolve the DNA in 9.5 mL Buffer EX until the DNA is completely dissolved (DNA should be dissolved by very gentle shaking. Avoid pipetting the DNA as this will cause shearing of large BAC/PAC/P1/Cosmid DNA).

15. Add 200 µL ATP-Dependent Exonuclease and 300 µL ATP solution to the dissolved DNA, mix gently but thoroughly, and incubate in a water bath or heating block at 37 °C for 60 min.
16. Equilibrate a QIAGEN-tip 500 by applying 10 mL Buffer QBT, and allow the column to empty by gravity flow.
17. Add 10 mL Buffer QS to the DNA sample from step 15, apply the whole sample to the QIAGEN-tip, and allow it to enter the resin by gravity flow.
18. Wash the QIAGEN-tip with 2 × 30 mL Buffer QC.
19. Elute DNA with 15 mL Buffer QF, pre-warmed to 65 °C
20. Precipitate DNA by adding 10.5 mL (0.7 volumes) room-temperature isopropanol to the eluted DNA. Mix and centrifuge immediately at ≥15,000× *g* for 30 min at 4 °C. Carefully decant the supernatant
(Alternatively is to centrifuge at 6000 rpm for 45 min at 4 °C in 50 mL FalconTube).
21. Wash DNA pellet with 5 mL room temperature 70% ethanol, and centrifuge at ≥15,000× *g* for 15 min. Carefully decant the supernatant without disturbing the pellet (alternative is to centrifuge at ,000 rpm for 15 min at 4 °C in 50 mL Falcon tube)
22. Air dry the pellet for 5–10 min, and dissolve the DNA in 50 µL sterile water. Use Nanospec to determine concentrations of DNA prep sample.

I. Minipreps of BAC Clones for Transfection—Modified from Qiagen

1. Grow cells o/n in 25 mL (50 mL tube) LB with antibiotics
Optional: Make 500 µL glycerol stock
2. Centrifuge culture at 6000 rpm 10 min
3. Discard supernatant and **completely** resuspend cells in 1000 µL Qiagen P1 or similar and divide equally into two Eppendorf tubes (e.g., 50 mM glucose, 25 mM Tris-HCl, pH 8, 10 mM EDTA, 100 µg/mL RNase)
4. Add 500 µL **P2 to each epp. tube** (Qiagen, 0.2 M NaOH, 1% SDS)—Mix by inverting until CLEAR (you can use the Qiagen blue dye to verify). **DO NOT VORTEX to avoid shearing.** Leave **MAX. 5 min** at room temperature
5. Add 500 µL **P3 each epp. tube** (Qiagen, 5 M K acetate: make by mixing 60 mL 5 M K acetate, 11.5 mL glacial acetic acid, and 28.5 mL water).
Mix completely by inverting. **DO NOT VORTEX to avoid shearing. Also avoids contamination with *E. coli* DNA.**

Optional: put on ice for 10 min (more solid precipitate forms?)

6. Centrifuge at 13,000 rpm at RT or 4 °C for min 10 min
7. Transfer **clear** supernatant in a separate 2 mL eppendorf (1 for **each epp. tube in step 1)** (If supernatant is not clear, pour in another tube and repeat step 6).
8. Add 1000 µL **isopropanol to each epp. tube** and mix well by inverting. Centrifuge at 13,000 rpm at RT or 4 °C for 30 min.
9. Pipet off supernatant; careful, do not remove the pellet

Small pellet: avoid using vacuum suction.

10. Add 500 µL **70% EtOH each epp. tube** (made with pure (dd) water), invert a few times to wash. Centrifuge at 13,000 rpm at RT for 10 min
11. Carefully remove all traces of ethanol without touching the pellet, then **air dry the pellet**,
12. Resuspend carefully in 95 µL water (by waiting and a few inversions; do not vortex)
13. **(If not already made from Qiagen large construct kit)**: Add 2 µL ATP-Dependent Exonuclease and 3 µL 100 mm ATP solution to the dissolved DNA, mix gently but thoroughly, and incubate in a water bath or heating block at 37 °C for 60 min.

14. Add an equal volume of phenol/chloroform and mix well but very gently to avoid shearing the DNA by inverting the tube until the phases are completely mixed. **CAUTION: PHENOL CAUSES SEVERE BURNS, WEAR GLOVES, GOGGLES, AND LAB COAT AND KEEP TUBES CAPPED TIGHTLY**
15. Carefully transfer the DNA/phenol mixture into a Phase Lock Gel™ tube and spin at 13,000 RPM for 5 min.
16. Transfer the upper aqueous phase to a new tube and add an equal volume of phenol/chloroform
17. Add 1/10 volume of sodium acetate. Mix well
18. Add 0.6 volumes of isopropanol and mix gently until the DNA precipitates.
19. Centrifuge at 13,000 rpm at RT or 4 °C for 20–30 min.
20. Pipet off supernatant; careful, do not remove the pellet

Small pellet: avoid using vacuum suction.

21. Add 500 μL **70% EtOH** (made with pure (dd) water), invert a few times to wash. Centrifuge at 13,000 rpm at RT for 10 min
22. Carefully remove all traces of ethanol without touching the pellet, then a**ir dry the pellet**
23. Resuspend carefully in 50–90 μL water (by waiting and a few inversions; do not vortex)

© 2016 by the authors; licensee MDPI, Basel, Switzerland. This article is an open access article distributed under the terms and conditions of the Creative Commons by Attribution (CC-BY) license (http://creativecommons.org/licenses/by/4.0/).
