# Supplementary material for: A Toolbox for Herpesvirus miRNA Research: Construction of a Complete Set of KSHV miRNA Deletion Mutants
Source: Viruses. 2016 Feb 19;8(2):54. doi: 10.3390/v8020054 (PMC4776209; doi:10.3390/v8020054)
Supplement: Supplementary file 1 [file viruses-08-00054-s001.zip › viruses-113147-supplementary (final)/Supplementary Table 1.pdf]

Supplementary Materials: A Toolbox for Herpesvirus miRNA Research: Construction of a Complete Set of KSHV miRNA Deletion Mutants

Vaibhav Jain, Karlie Plaisance-Bonstaff, Rajnikumar Sangani, Curtis Lanier, Alexander Dolce, Jianhong Hu, Kevin Brulois, Irina Haecker, Peter Turner, Rolf Renne and Brian Krueger

Table S1. KSHV miRNA sequences and name conversion table.

miRNA Name	miRNA Sequence	Coordinates in GQ994935 (BAC16)	Samols *et al.*, 2005; * Grundhoff *et al.*, 2006	Pfeffer *et al.*, 2005	Cai *et al.*, 2005	miRBase Name mirbase.org
miR-K12-1	AUUACAGGAAACUGGGUGUAAGC	122,354–122,332	KSHV-miRNA-1	miR-K12-1	miR-K1	kshv-miR-K12-1-5p
miR-K12-2	AACUGUAGUCCGGGUCGAUCUG	122,193–122,172	-	-	miR-K2	kshv-miR-K12-2-5p
miR-K12-3	UCACAUUCUGAGGACGGCAGCG	122,051–122,030	KSHV-miRNA-2	miR-K12-3 (5p)	miR-K3	kshv-miR-K12-3-5p
	UCGCGGUCACAGAAUGUGACA	122,009–121,989	-	miR-K12-3 (3p)	-	kshv-miR-K12-3-3p
miR-K12-4	AGCUAAACCGCAGUACUCUAGG	121,919–121,898	KSHV-miRNA-3-5p	miR-K12-4 (5p)	miR-K4-5p	kshv-miR-K12-4-5p
	UAGAAUACUGAGGCCUAGCUGA	121,881–121,860	KSHV-miRNA-3-3p	miR-K12-4 (3p)	miR-K4-3p	kshv-miR-K12-4-3p
miR-K12-5	UAGGAUGCCUGGAACUUGCCGG	121,730–121,709	KSHV-miRNA-4	miR-K12-5	miR-K5	kshv-miR-K12-5-3p
	CCAGCAGCACCUAAUCCAUCGG	121,260–121,239	-	miR-K12-6 (5p)	miR-K6-5p	kshv-miR-K12-6-5p
miR-K12-6	UGAUGGUUUUCGGGCUGUUGAG	121,228–121,207	KSHV-miRNA-5	miR-K12-6 (3p)	miR-K6-3p	kshv-miR-K12-6-3p
miR-K12-7	UGAUCCCAUGUUGCUGGCGCU	122,822–122,802	KSHV-miRNA-7	miR-K12-7	miR-K7	kshv-miR-K12-7-3p
	CUCCCUCACUAACGCCCCGC	120,448–120,429	-	-	miR-K8-5p	kshv-miR-K12-8-5p
miR-K12-8	UAGGCGCGACUGAGAGAGCACG	120,408–120,387	KSHV-miRNA-8	miR-K12-8	miR-K8-3p	kshv-miR-K12-8-3p
miR-K12-9	ACCCAGCUGCGUAAACCCCGCU	119,801–119,780	-	miR-K12-9 (5p)	miR-K9-5p	kshv-miR-K12-9-5p
	CUGGGUAUACGCAGCUGCGUAA	119,767–119,746	KSHV-miRNA-9	miR-K12-9 (3p)	miR-K9-3p	kshv-miR-K12-9-3p
miR-K12-10	UAGUGUUGUCCCCCCGAGUGGC	117,810–117,789	KSHV-miRNA-10	miR-K12-10a	miR-K10	kshv-miR-K12-10a-3p
	U**G**GUGUUGUCCCCCCGAGUGGC	Not present	-	miR-K12-10b	-	kshv-miR-K12-10b
miR-K12-11	UUAAUGCUUAGCCUGUGUCCGA	121,043–121,022	KSHV-miRNA-6	miR-K12-11	-	kshv-miR-K12-11-3p
miR-K12-12	AACCAGGCCACCAUUCCUCUCCG	117,570–117,548	*miR-K12-12	-	-	kshv-miR-K12-12-5p

© 2016 by the authors; licensee MDPI, Basel, Switzerland. This article is an open access article distributed under the terms and conditions of the Creative Commons by Attribution (CC-BY) license (http://creativecommons.org/licenses/by/4.0/).
