# Supplementary material for: A Toolbox for Herpesvirus miRNA Research: Construction of a Complete Set of KSHV miRNA Deletion Mutants
Source: Viruses. 2016 Feb 19;8(2):54. doi: 10.3390/v8020054 (PMC4776209; doi:10.3390/v8020054)
Supplement: Supplementary file 1 [file viruses-08-00054-s001.zip › viruses-113147-supplementary (final)/Supplementary Table 2.pdf]

Supplementary Materials: A Toolbox for Herpesvirus miRNA Research: Construction of a Complete Set of KSHV miRNA Deletion Mutants

Vaibhav Jain, Karlie Plaisance-Bonstaff, Rajnikumar Sangani, Curtis Lanier, Alexander Dolce, Jianhong Hu, Kevin Brulois, Irina Haecker, Peter Turner, Rolf Renne and Brian Krueger

Supplementary Table 2. Primers used in mutant construction

The coordinates given are for the locations in the BAC16 genome (GQ994935). Forward primers are indicated by "F" and reverse by "R". The underlined portions in a primer pair are complementary to one another.

Deletion Primers for miR-K12-1 Deleted for bases 122,332-122,354

	122,292-122,311	122,312-122,331	122,355-122,374	Kan 5'
F	GAGGGTTGCAGGAAACAGGT	GCTGCCGGGGATTATGTACA	CGCCCGCATCCGGCCGTCCT	AGGATGACGACGATAAGTAGGG
	122,394-122,375	122,374-122,355	122,331-122,312	Kan 3'
R	CGCAGGGTGCGGTGCTGCCC	AGGACGGCCGGATGCGGGCG	TGTACATAATCCCCGGCAGC	AACCAATTAACCAATTCTGATTAG

Deletion Primer for miR-K12-2 Deleted for bases 122,172-122,193

	122,132 – 122,151	122,152 – 122,171	122,194 – 122,213	Kan 5'
F	GCTCTAGCCCTGGAAGATCT	GGAAGCTTGCTTCAATGGCT	AGCGAAGTAGACCCATTATA	AGGATGACGACGATAAGTAGGG
	122,233 – 122,214	122,213 - 122,194	122,171 - 122,152	Kan 3'
R	ACCAGATTTAAGATTAAGAT	TATAATGGGTCTACTTCGCT	AGCCATTGAAGCAAGCTTCC	AACCAATTAACCAATTCTGATTAG

Deletion Primers for miR-K12-3 Deleted for bases 122,030-122,051

	121,990-122,009	122,010-122,029	122,052-122,071	Kan 5'
F	GTCACATTCTGTGACCGCGA	CGTTGACGTTAGACACACGT	TAGCCCATTACAGGCATTGT	AGGATGACGACGATAAGTAGGG
	122,091-122,072	122,071-122,052	122,029-122,010	Kan 3'
R	TCCCAACCAACGCAACAGCT	ACAATGCCTGTAATGGGCTA	ACGTGTGTCTAACGTCAACG	AACCAATTAACCAATTCTGATTAG

Deletion Primers for miR-K12-4 Deleted for bases 121,898-121,919

	121,858-121,877	121,878-121,897	121,920-121,939	Kan 5'
F	AATCAGCTAGGCCTCAGTAT	TCTATGTAACAAATGAATGC	AGTTATACTGCCCGGTTCCA	AGGATGACGACGATAAGTAGGG
	121,959-121,940	121,939-121,920	121,897-121,878	Kan 3'
R	GTGGCCACAACGCCGCCTGG	TGGAACCGGGCAGTATAACT	GCATTCATTTGTTACATAGA	AACCAATTAACCAATTCTGATTAG

Deletion Primers for miR-K12-5 Deleted for bases 121,709-121,730

	121,669–121,688	121,689–121,708	121,731–121,750	Kan 5'
F	TTTTTGTGGCACCAGGTTAT	GGTCTTATGAGCGGGCTTGA	AGTGCTTGATGTAGACCCTT	AGGATGACGACGATAAGTAGGG
	121,770–121,751	121,750-121,731	121,708–121,689	Kan 3'
R	TAGGTAGTCCCTAGTGCCCT	AAGGGTCTACATCAAGCACT	TCAAGCCCGCTCATAAGACC	AACCAATTAACCAATTCTGATTAG

Deletion Primers for miR-K12-6 Deleted for bases 121,207-121,228

	121,167-121,186	121,187-121,206	121,229-121,248	Kan 5'
F	ACACACAGAACAATAACGGG	CGACTAGATAAAAAGACTCG	GCCCGCACCGCCGATGGATT	AGGATGACGACGATAAGTAGGG

	121,268-121,249	121,248-121,229	121,206-121,187	Kan 3'
R	AGACTTGTCCAGCAGCACCT	AATCCATCGGCGGTGCGGGC	CGAGTCTTTTTATCTAGTCG	AACCAATTAACCAATTCTGATTAG

Deletion Primers for miR-K12-7 Deleted for bases 120,801-120,822

	120,761-120,780	120,781-120,800	120,823-120,842	Kan 5'
F	TGCGTGCTTTCGACGTCCAG	GCGGCTGGCACACGGGCCGT	TGGTAGTAAGATACAGCATA	AGGATGACGACGATAAGTAGGG

	120,862-120,843	120,842-120,823	120,800-120,781	Kan 3'
R	AGCGCCACCGGACGGGGATT	TATGCTGTATCTTACTACCA	ACGGCCCGTGTGCCAGCCGC	AACCAATTAACCAATTCTGATTAG

Deletion Primers for miR-K12-8 Deleted for bases 120,388-122,409

	120,348-120,367	120,368-120,387	120,410-120,429	Kan 5'
F	TGTTGGCCGCGTGCCAGGCG	CCGGCGTGGGCGCCGCGCGC	CTGCTTCCAACAGACAAAAG	AGGATGACGACGATAAGTAGGG

	120,449-120,430	120,429-120,410	120,387-120,368	Kan 3'
R	ACTCCCTCACTAACGCCCCG	CTTTTGTCTGTTGGAAGCAG	GCGCGCGGCGCCCACGCCGG	AACCAATTAACCAATTCTGATTAG

Deletion Primers for miR-K12-9 Deleted for bases 119,780-119,801

	119,740-119,759	119,760-119,779	119,802-119,821	Kan 5'
F	CCGGGTTTACGCAGCTGCGT	ATACCCAGCTGTGTTTACGC	AGACCCAGCTGGGTATACCT	AGGATGACGACGATAAGTAGGG

	119,841-119,822	119,821-119,802	119,779-119,760	Kan 3'
R	ATCGCAGCCCCTATTCCAGT	AGGTATACCCAGCTGGGTCT	GCGTAAACACAGCTGGGTAT	AACCAATTAACCAATTCTGATTAG

Mutation Primers for miR-K12-10

WILD TYPE
117,789-117,811
GCCACTCGGGGGGACAACACTAA

	117,749-117,768	117,769-117,788	MUTANT	117,812-117,831
F	CCGTTGCAACTCGTGTCCTG	AATGCTACGGGGCCACGCTG	GCCA**GAA**GG**A**GG**A**AC**C**ACA**GA**AA	TCGCCAACAGACAAACGAGT

Kan 5'
AGGATGACGACGATAAGTAGGG

	117,851-117,832	117,831-117,812	MUTANT	117,788-117,769
R	AGGCTTGGGGCGATACCACC	ACTCGTTTGTCTGTTGGCGA	TT**TC**TGT**G**GT**T**CC**T**CC**TTC**TGGC	CAGCGTGGCCCCGTAGCATT
			I S V V P P S G	

Kan 3'
AACCAATTAACCAATTCTGATTAG

WILD TYPE
117,811-117,789
TTAGTGTTGTCCCCCCGAGTGGC
I S V V P P S G

Deletion Primers for miR-K12-11 Deleted for bases 121,022-121,043

```
  120,982-121,001      121,002-121,021      121,044-121,063      Kan 5'
F TCGGGAACCCGCCCGCGCTT CCGCAAGGCACGGGGCCGCA GGATCATAACACCGCCCTAG AGGATGACGACGATAAGTAGGG

  121,083-121,064      121,063-121,044      121,021-121,002      Kan 3'
R TGGTCACAGCTTAAACATTT CTAGGGCGGTGTTATGATCC TGCGGCCCCGTGCCTTGCGG AACCAATTAACCAATTCTGATTAG
```

Deletion Primers for miR-K12-12 Deleted for bases 117,547-117,572

```
  117,612-117,593      117,592-117,573      117,546-117,527      Kan 5'
F CTCATAACACAGTCACAGTT CAGAAGGCCGGCACGCGGTG ATTAAAGCACTCGGTGGGGG AGGATGACGACGATAAGTAGGG

  117,507-117,526      117,527-117,546      117,573-117,592      Kan 3'
R TGTGTCAACCAGGGCACCCT CCCCCACCGAGTGCTTTAAT CACCGCGTGCCGGCCTTCTG AACCAATTAACCAATTCTGATTAG
```

Cluster deletion Deleted for bases 119,780-122,354 spanning miR-K12-1 through miR-K12-9, and miR-K12-11

```
   119,740-119,759      119,760-119,779      122,355-122,374      Kan 5'
9F CCGGGTTTACGCAGCTGCGT ATACCCAGCTGTGTTTACGC CGCCCGCATCCGGCCGTCCT AGGATGACGACGATAAGTAGGG

   122,394-122,375      122,374-122,355      119,779-119,760      Kan 3'
1R CGCAGGGTGCGGTGCTGCCC AGGACGGCCGGATGCGGGCG GCGTAAACACAGCTGGGTAT AACCAATTAACCAATTCTGATTAG
```

© 2016 by the authors; licensee MDPI, Basel, Switzerland. This article is an open access article distributed under the terms and conditions of the Creative Commons by Attribution (CC-BY) license (http://creativecommons.org/licenses/by/4.0/).
